# Development of loop-mediated isothermal amplification (LAMP) assays using five primers reduces the false-positive rate in COVID-19 diagnosis

**DOI:** 10.1038/s41598-023-31760-z

**Published:** 2023-03-28

**Authors:** Galyah Alhamid, Huseyin Tombuloglu, Ebtesam Al-Suhaimi

**Affiliations:** 1 Master Program of Biotechnology, Institute for Research and Medical Consultations, Imam Abdulrahman Bin Faisal University, Dammam, 31441 Saudi Arabia; 2 Department of Genetics Research, Institute for Research and Medical Consultations (IRMC), Imam Abdulrahman Bin Faisal University, Dammam, 31441 Saudi Arabia; 3 Biology Department, College of Science and Institute of Research and Medical Consultations (IRMC), Imam Abdulrahman Bin Faisal University, Dammam, 31441 Saudi Arabia

**Keywords:** SARS-CoV-2, Infectious-disease diagnostics, Virology

## Abstract

The reverse-transcription loop-mediated isothermal amplification (RT-LAMP) is a cheaper and faster testing alternative for detecting SARS-CoV-2. However, a high false-positive rate due to misamplification is one of the major limitations. To overcome misamplifications, we developed colorimetric and fluorometric RT-LAMP assays using five LAMP primers, instead of six. The gold-standard RT-PCR technique verified the assays' performance. Compared to other primer sets with six primers (N, S, and RdRp), the E-ID1 primer set, including five primers, performed superbly on both colorimetric and fluorometric assays. The sensitivity of colorimetric and fluorometric assays was 89.5% and 92.2%, respectively, with a limit of detection of 20 copies/µL. The colorimetric RT-LAMP had a specificity of 97.2% and an accuracy of 94.5%, while the fluorometric RT-LAMP obtained 99% and 96.7%, respectively. No misamplification was evident even after 120 min, which is crucial for the success of this technique. These findings are important to support the use of RT-LAMP in the healthcare systems in fighting COVID-19.

## Introduction

The coronavirus disease (COVID-19) is caused by severe acute respiratory syndrome coronavirus 2 (SARS-CoV-2) and continues to cause rapid infections and deaths since its emergence around 3 years ago. The virus enters the body through the respiratory system, infecting multiple organs and causing damage to multiple organ systems^1^. As of February 2023, the global number of cases reached 675 million and the death toll exceeded 6.7 million^2^. However, the emergence of new variants with high mutation rates causes increased infectivity and decreased effectiveness of vaccines. This hinders the efficient control of this pandemic. To diagnose this disease, reverse-transcription polymerase chain reaction (RT-PCR) is the accepted gold standard technique. However, it requires sophisticated equipment, a laboratory environment, and expert personnel to perform the tests. These requirements prevent rapid and expanded testing, especially in regions with lower resources. Therefore, researchers are investigating alternative and affordable point-of-care (POC) testing methods that can diagnose COVID-19 with high sensitivity.

Isothermal nucleic acid amplification techniques are becoming more attractive because they do not require a thermocycler, hence, they are cheaper than RT-PCR with comparable sensitivity. Several rapidly-advancing isothermal amplification techniques were developed for pathogen detection, such as rolling circle amplification (RCA)^3^, recombinase polymerase amplification (RPA)^4^, strand-displacement amplification (SDA)^5^, ladder-shape melting temperature isothermal amplification (LMTIA)^6^, and reverse-transcription loop-mediated isothermal amplification (RT-LAMP)^7^. RT-LAMP uses four to six primers to target and amplify a specific gene region^8^. The amplification reaction in this technique takes place isothermally in a single tube without requiring bulky instruments. It provides simple detection techniques, being also cheaper and faster than the gold standard^7,9,10^. The efficiency of RT-LAMP in COVID-19 diagnosis was demonstrated by many researchers^11–16^, and many validated the performance of FDA-approved colorimetric RT-LAMP kits for emergency use authorization^17,18^. However, because of the number of primers, the most common limitation of the RT-LAMP technique is the misamplifications that arise from unwanted secondary structures. Even with the careful primer design and the availability of programs that check for dimer and hairpin structures, there is no guarantee that these structures will not be formed practically. In particular, current protocols recommend that the reaction time must not exceed 30 min to avoid misleading results^11,14,19^. This makes it challenging to detect samples with low copy numbers and reduces the efficiency of the RT-LAMP technique.

To address this issue, we hypothesize that a lower number of LAMP primers will reduce the false positivity rate, which is the most common limitation of the LAMP technique^20,21^. The slower amplification rate can be improved by optimizing the performance of the developed colorimetric and fluorometric assays via increasing the enzyme’s concentration or adding primer binding enhancers like guanidine hydrochloride (GuHCl), as shown elsewhere^22–24^. In this work, we demonstrate the efficiency of using five primers to reduce misamplifications by comparing them with five primer sets targeting different viral genes, such as *RdRP*, *S*, and *N*. The optimized protocol using five primers (E-ID1) eliminates the misamplifications, thus improving the detection’s sensitivity and efficacy. By the time of writing this manuscript, no studies discussed the efficiency of primer sets with five primers in the colorimetric and fluorometric detection of SARS-CoV-2, which will be comprehensively addressed here.

## Results

### RT-LAMP primers performance

**Figure 1:**
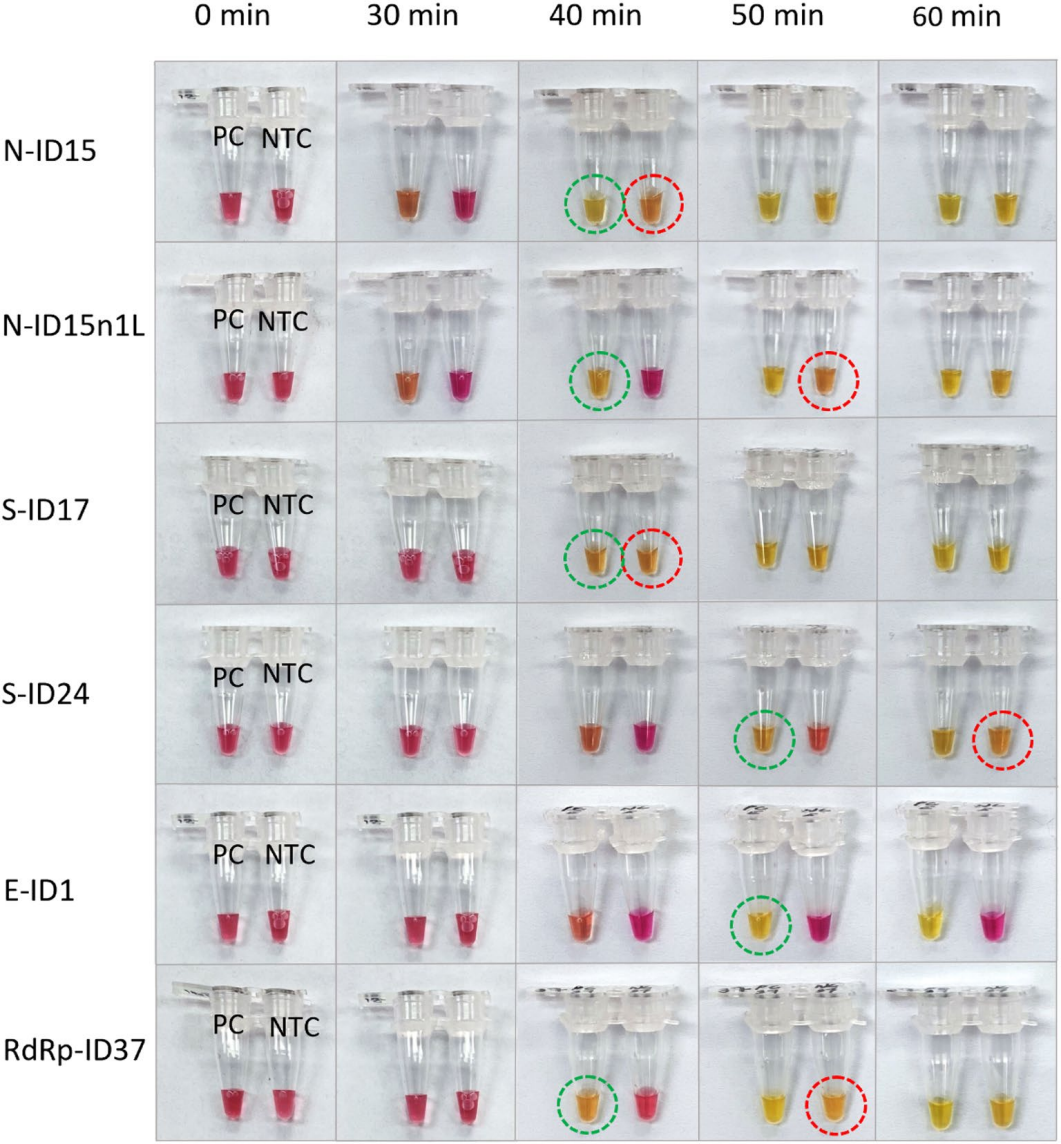
Colorimetric test using all primer sets on positive controls (PC) and non-template controls (NTC). A visible color change from red to yellow develops in PC tubes in the colorimetric identification, and negatives remain red. The color development was observed before (0 min) and after the reaction (between 30 and 60 min). The green circle denotes the earliest color development in the PC reaction tubes. The red circles represent the NTC reactions that led to the earliest misamplification.

The performance of the six sets of primers targeting different genes like *N*, *E*, *S*, or *RdRp* was tested on positive (PC) and non-template controls (NTC). In the colorimetric identification, the color in all tubes was observed at before the reaction (0 min) and then every 10 min. According to the results shown in Fig. 1, primer sets N-ID15n1L and RdRp-ID37 showed a red-to-yellow color difference in positive reactions at 40 min. Primer set E-ID1 started to develop a color change at 50 min (without optimization), while most primer sets began to show misamplifications, as the color changed to yellow in NTC tubes. The reaction was terminated after 60 min due to developing false positivity in NTC in all the primer sets except E-ID1.

Depending on the primer set, the appearance time of false-positive results differs. Since misamplification is evident in the NTC tubes of N-ID15 and S-ID17 for around 40 min, it is not recommended to incubate the reaction for longer than 40 min. However, extending the time over 120 min did not lead to misamplifications for the E-ID1 set harboring five primers. Therefore, the E-ID1 primer set was selected for further testing.

### Optimization of the colorimetric and fluorometric RT-LAMP assays

**Figure 2:**
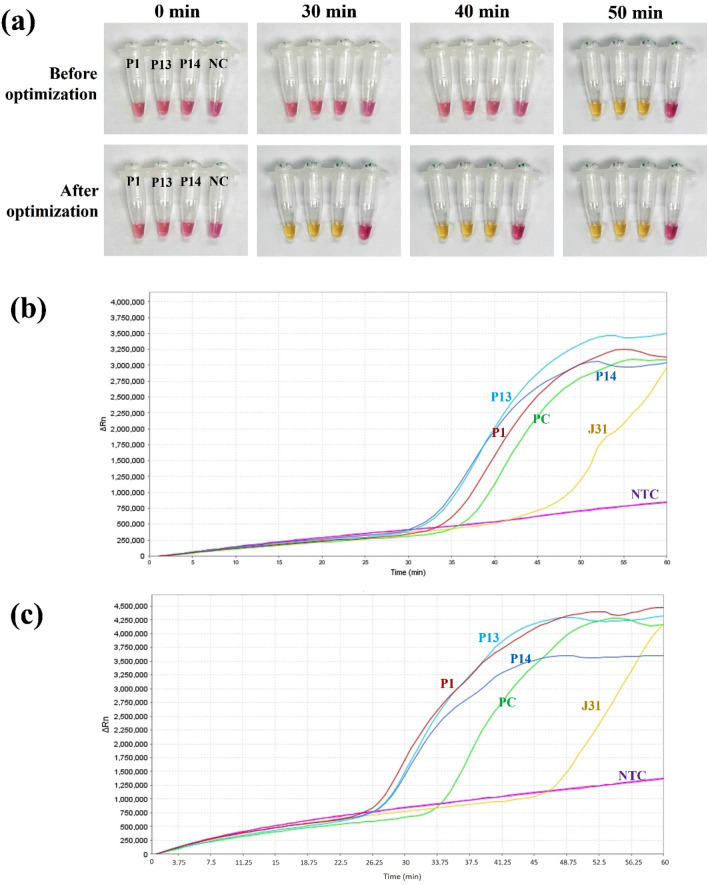
(**a**) Colorimetric RT-LAMP using E-ID1 primer set on clinical samples before and after optimization. The optimized colorimetric assay started to detect the positive samples within 30 min compared to 50 min before optimization. (**b**) Fluorometric RT-LAMP before (cycle number on the x-axis also corresponds to reaction time, in minutes, since the reaction set up here was 60 cycles of 1 min) and (**c**) after optimization using LavaLAMP reagents (Lucigen, Middleton, WI, USA) on different clinical samples, where the improvement in the detection time can be evident.

Colorimetric and fluorometric RT-LAMP assays were developed and optimized to enhance the performance of the *E* gene primers. The optimization includes (1) the addition of guanidine hydrochloride (GuHCl), (2) testing different DNA polymerases (Bst 2.0 *vs* Bst 3.0 versions), and (3) adjusting the optimum reaction temperature. In contrast to the detection time before optimization (~ 50 min), the colorimetric assay showed an earlier color change within 30 min (Fig. 2a). Also, this improvement was seen in the fluorometric RT-LAMP assay, where it detected the positive samples (P1, P13, P14, and PC) on an average of around 27 min, compared to the average detection time of 32 min before the optimization (Fig. 2b,c). There was no improvement in the detection time of sample J31 in this run, however, several experiments showed that J31 was undetected when using different enzyme concentrations (Table S1).

Compared to Bst 2.0 DNA polymerase, Bst 3.0 DNA polymerase enzyme (New England Biolabs (NEB), USA) is designed to have a higher amplification performance and reverse transcriptase activity, allowing single enzyme RT-LAMP reactions https://international.neb.com/products/m0374-bst-3-0-dna-polymerase#Product%20Information. By comparing with Bst 2.0 DNA polymerase in the fluorometric RT-LAMP, the enzyme that shows the best performance with the E-ID1 primer set was chosen for further analysis. Both enzymes were added at the same concentration on PC and NTC triplicates. However, positive amplifications appeared around 17 min using Bst 2.0 DNA polymerase enzyme, while with Bst 3.0 DNA polymerase, PC amplified after 21 min (Figure S1). This result revealed that Bst 2.0 DNA polymerase enzyme performs faster than Bst 3.0 DNA polymerase.

**Figure 3:**
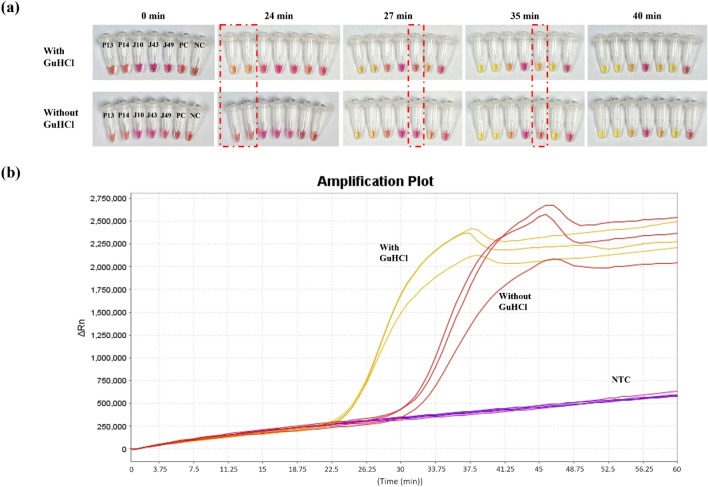
Testing the effect of adding 40 mM guanidine hydrochloride (GuHCl) in (**a**) colorimetric RT-LAMP reaction on positive clinical samples (P13, P14, J10, J49) and an inconclusive sample (J43). This addition improved the detection time in one sample (J49), where it started to change color 8 min earlier compared to reactions without GuHCl. The inconclusive sample J43, however, remained negative in both. The red dashed rectangle highlights the samples having an earlier color change to yellow with the GuHCl addition at the same reaction time. (**b**) Fluorometric RT-LAMP PC and NTC triplicate test with and without GuHCl. All PC triplicates with GuHCl amplified earlier than those without. There were no amplifications in NTC in both colorimetric and fluorometric tests, suggesting that GuHCl enhances the detection speed without causing misamplification in the NTC.

The effect of GuHCl on the performance of the E-ID1 primer set in both colorimetric and fluorometric RT-LAMP reactions was tested since it was reported to enhance the detection speed and efficiency^22–24^. Figure 3a shows two sets of colorimetric RT-LAMP with and without adding GuHCl (40 mM) on positive clinical specimens, PC, and NTC. The colors started to develop in the positive samples after 24–27 min. In reactions with GuHCl, sample J49 began to show a color change after 27 min, and the yellow color fully developed after 30 min. On the other hand, this sample’s color started to change after 35 min without adding GuHCl, which was fully developed after 40 min. The effect of GuHCl was also tested fluorometrically on PC and NTC triplicates (Fig. 3b). The mean detection time of the PC reactions without GuHCl was 29 min, while it was about 22.5 min with the GuHCl addition, which corresponds to a 22% improvement in the detection time. No misamplification in either test was evident upon 120 min.

### Limit of detection of the *E* gene primers

**Figure 4:**
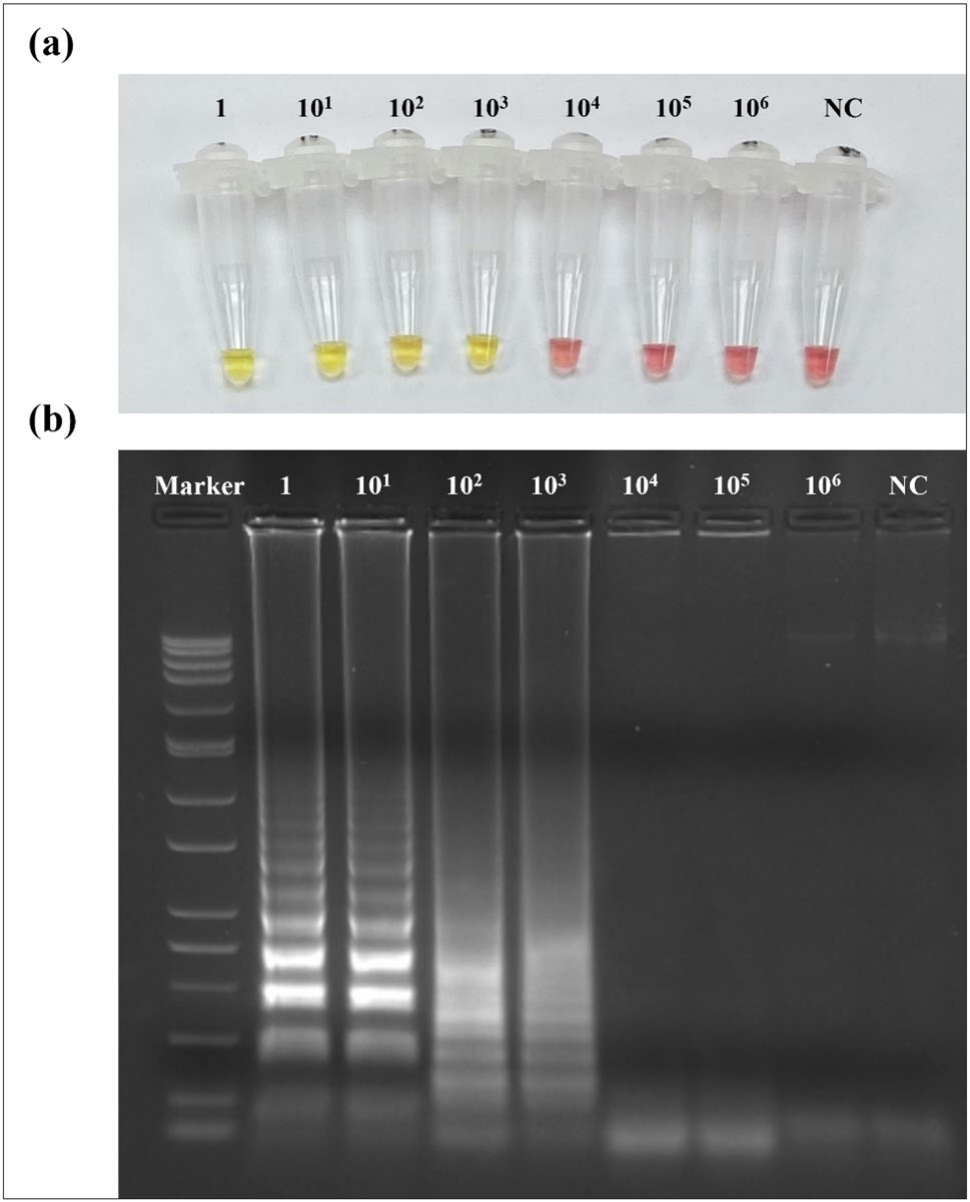
The limit of detection (LoD) of *E* gene primers tested in the colorimetric RT-LAMP assay on serially diluted SARS-CoV-2 synthetic RNA control (**a**). The assay detected up to 10^3^ dilutions corresponding to 500 copies/reaction volume (25 µL) or 20 copies/µL. (**b**) Validation of the colorimetric LoD results via loading the RT-LAMP product post-reaction in 2% agarose gel showing band patterns of the detected diluted samples.

SARS-CoV-2 synthetic RNA control (Twist synthetic RNA control 51 (EPI_ISL_7718520), Twist Bioscience, USA) was serially diluted (1, 10^1^, 10^2^, 10^3^, 10^4^, 10^5^, and 10^6^ times) to determine the limit of detection (LoD) of RT-LAMP reactions. Figure 4a shows the LoD of the colorimetric RT-LAMP assay targeting the *E* gene. The color development to yellow is prominent in 1, 10^1^, 10^2^, and 10^3^-times diluted RNA, corresponding to 500 copies/reaction volume (25 µL) or 20 copies/µL for the 10^3^-times dilution. After the reaction, the reaction mixture of each dilution was loaded in 2% agarose and visualized under a UV trans-illuminator to check further if band-pattern intensities decreased with reduced viral loads (Fig. 4b). The results indicate a ladder-type banding pattern in the detected dilutions, while no bands appeared in the others. Both agarose gel electrophoresis and colorimetric identifications were in line.

### RT-LAMP assays on clinical samples

**Figure 5:**
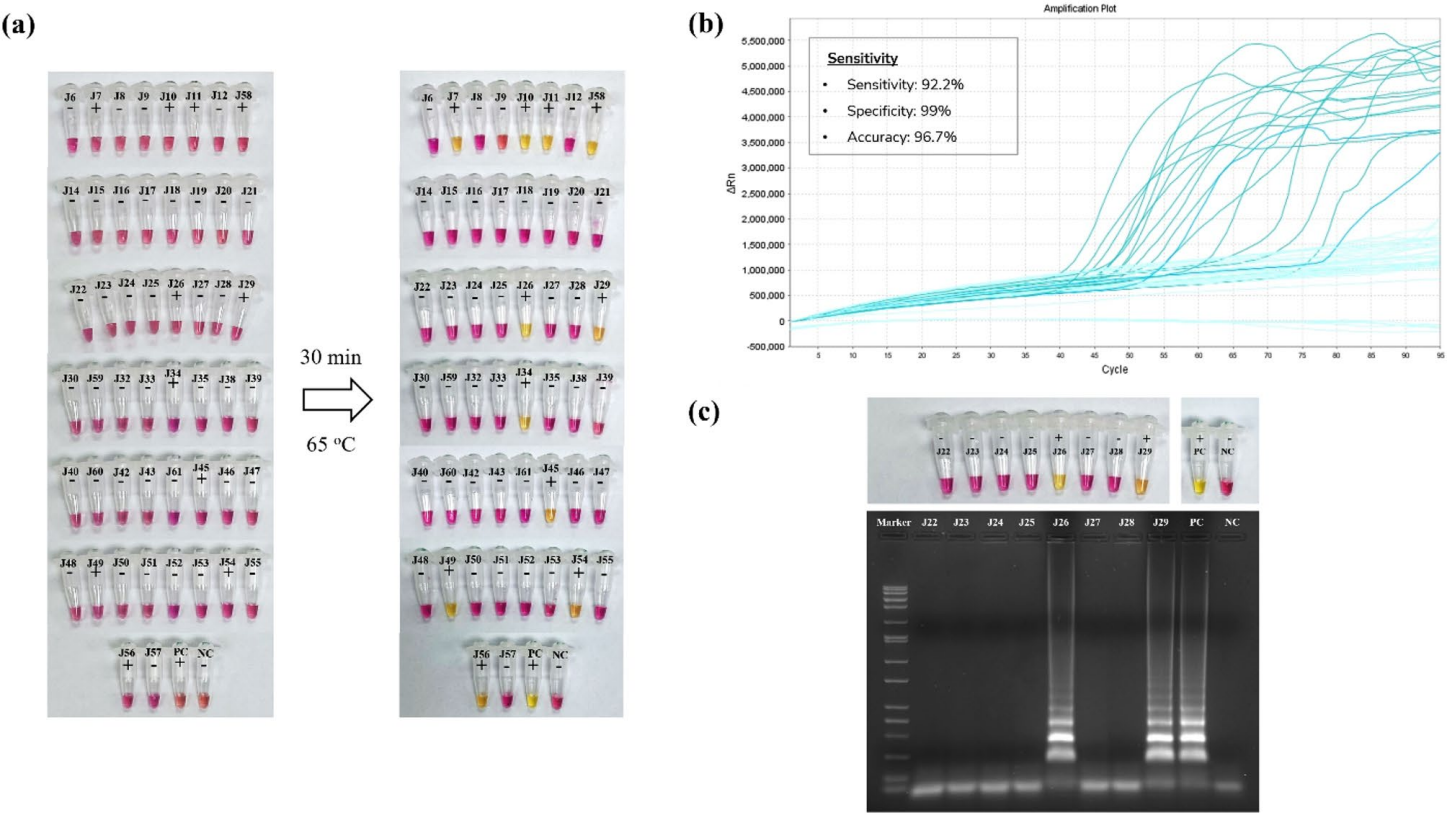
Optimized (**a**) colorimetric and (**b**) fluorometric RT-LAMP assays tested on SARS-CoV-2 clinical samples show agreement in the detection sensitivity. The sample codes of each amplification curves are provided in Figure S7. (**c**) Loading eight samples in 2% agarose stained with a DNA stain, a 0.05–10 Kb DNA ladder was used as a marker, where positive samples showed DNA bands under a UV transilluminator.

The performance of the E-ID1 primer set was colorimetrically and fluorometrically tested on clinical specimens, as depicted in Fig. 5a. These results were then validated using agarose gel electrophoresis (Fig. 5c). In the colorimetric RT-LAMP reaction, the tubes were incubated in a thermal block set at 65 °C. The positive clinical specimens started to show a color change after 20 min, while the color difference between positives and negatives was the clearest after 30 min. Eventually, there was 94.5% agreement between the colorimetric RT-LAMP and RT-PCR results.

Similarly, clinical samples were fluorometrically tested using the optimized RT-LAMP protocol (Fig. 5b). The reaction was placed at 70 °C in a thermal cycler for 75 min. This fluorometric assay had 96.7% agreement with RT-PCR results.

### Sensitivity and specificity of RT-LAMP assays

In the colorimetric RT-LAMP assay, a total of 165 clinical specimens were tested (57 positive and 108 negative samples, according to RT-PCR results) (Table S3). With reference to RT-PCR, 51 out of 57 samples agreed as positive, and 105 out of 108 were negative, with three false positives and six false negative results. Accordingly, the colorimetric RT-LAMP assay had a sensitivity of 89.5%, specificity of 97.2%, and accuracy of 94.5%. The positive percent value (PPV) with the RT-PCR was calculated to be 94.4%, while the negative percent value (NPV) was 94.6% (Table S4). In the fluorometric detection, 150 clinical specimens were tested, 51 of which were RT-PCR-positive and 99 were RT-PCR-negative. 47 out of 51 positive and 98 out of 99 negative results agreed with RT-PCR, with one false positive and four false negative results. The fluorometric assay had 92.2% sensitivity, 99% specificity, and 96.7% accuracy. The fluorometric RT-LAMP assay had a PPV of 98% and an NPV of 96.1% (Table S3, S4).

**Figure 6:**
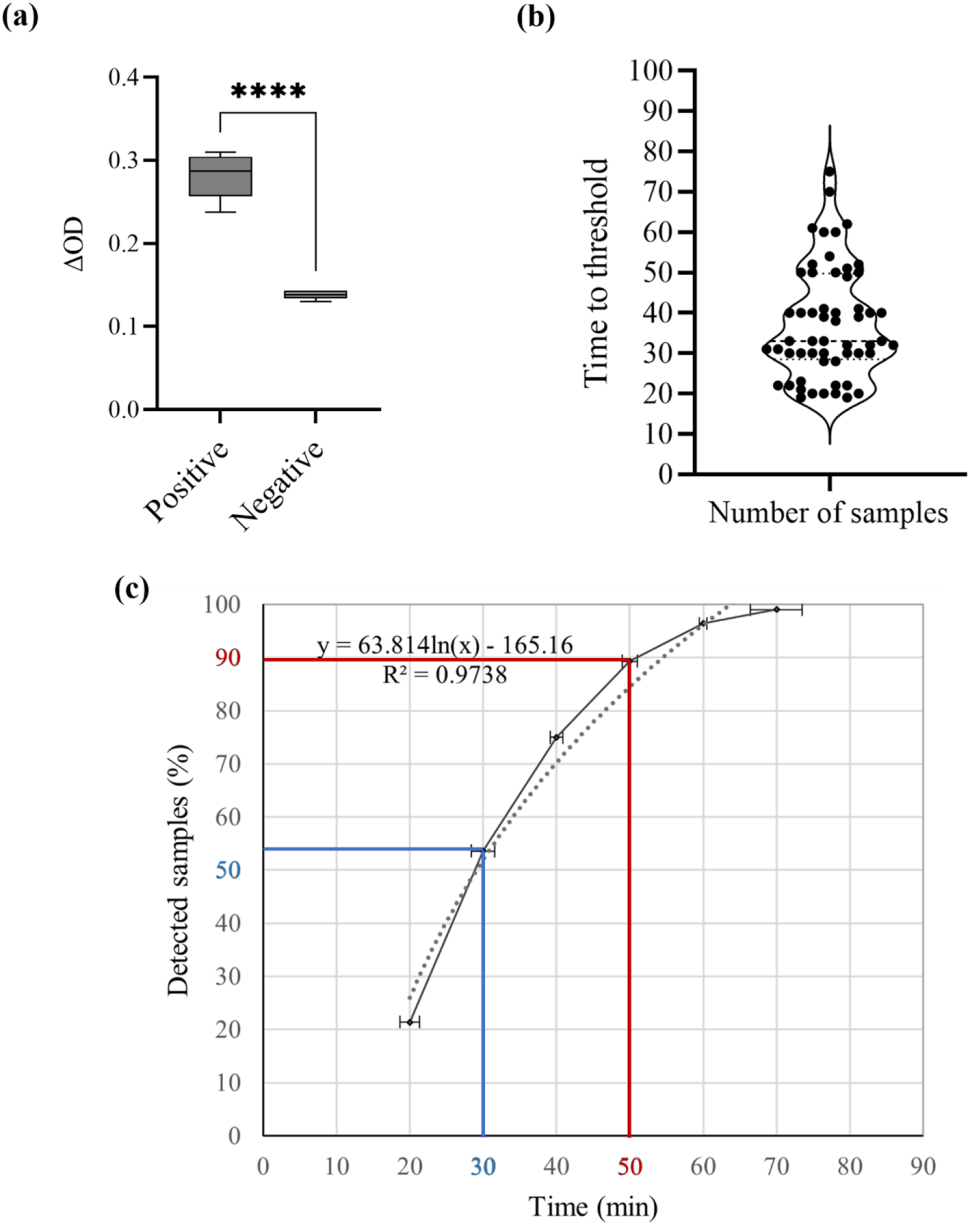
(**a**) Quantification of the color change between positive and negative samples by the spectrophotometric measurement of optical density (ΔOD) at 434 and 560 nm (n = 10). The line inside the box represents the median, and the whiskers extend to the maximum and minimum values. The four asterisks (*) correspond to *p* < 0.0001, which is a statistically significant difference (unpaired *t* test, *p* < 0.05, 95% CI). (**b**) Time to the detection threshold of all positive samples. The dashed line indicates the median detection time (33 min). (**c**) Percentage of total positive samples detected plotted against time. All samples (100%) were detected into 75 min. In addition, 90% of the SARS-CoV-2 positive specimens were diagnosed in 50 min.

The color development in the positive and negative samples was quantified post-reaction using the spectrophotometer by measuring the absorbance at 434 and 560 nm wavelengths. Figure 6a shows that the difference in optical densities (ΔOD) between the positive yellow and negative red samples is statistically significant (*p* < 0.0001, 95% CI). Among the tested samples, the samples having high viral loads were detected in ~ 20 min, while those with low viral loads took up to 75 min (Fig. 6b,c). On average, the detection time was calculated as 37 min. Besides, 90% of SARS-CoV-2 positive samples were detected within 50 min (Fig. 6c). All high, medium, and low viral load samples were included in the diagnostic reliability calculations (Table S3, S4).

In addition, the inclusivity of the E-ID1 primer set was tested in-silico by aligning five SARS-CoV-2 variants with other SARS viruses. The primers bind to the conserved regions in all the variants but are uncommon for the other SARS (Figure S3). In addition, the cross-reactivity of E-ID1 primers was tested in-vitro against the most common respiratory viruses, including parainfluenza virus 3, enterovirus, rhinovirus, human metapneumovirus A + B, parainfluenza virus 4, bocavirus, and coronavirus 229 E. The colorimetric assay only detected SARS-CoV-2 but none of the other respiratory viruses, which indicates high specificity against the diagnosis of COVID-19 infection (Figure S4).

### Using five primers delays or prevents misamplification (false-positives) in all primer sets

**Figure 7:**
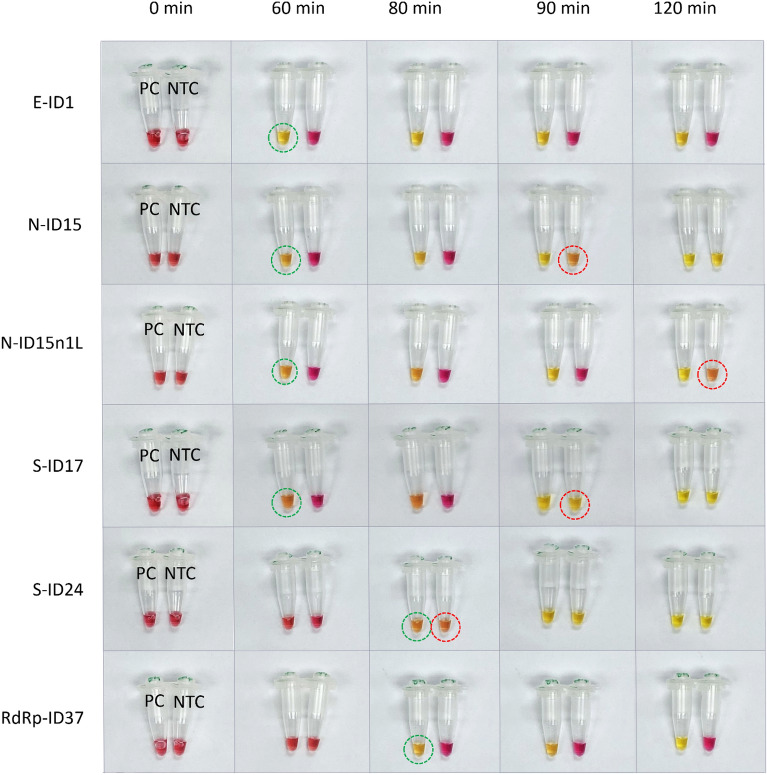
Colorimetric test using all primer sets, each containing five primers (F3, B3, FIP, BIP, and LB) on Omicron positive controls (PC) and non-template controls (NTC). All primer sets show a higher tolerance for longer reaction times before producing misamplifications compared to using the same primer sets with six primers (Fig. 1). However, N-ID15, N-ID15n1L, S-ID17, and S-ID24 primer sets (without the LF primer) still produced misamplifications in NTC between 90 and 120 min. Reversely, the false-positive amplification was not evident by using the E-ID1 and RdRp-ID37 primers even up to 120 min.

All primer sets were tested on PC and NTC samples to confirm the `five-primer advantage` in preventing false-positive results. Figure 7 compares the performance of the E-ID1 set (after optimization) and the other previously tested primer sets (N-ID15, N-ID15n1L, S-ID17, S-ID24, and RdRp-ID37) prepared with five primers instead of six (without LF primer). A delayed misamplification was evident for N-ID15, N-ID15n1L, S-ID17, and S-ID24 sets compared to their initial performance with six primers as shown in Fig. 1. In addition, the color change was observed and recorded for 120 min. Using five primers in all primer sets tolerated longer reaction time without false amplification. Furthermore, primer sets for E-ID1 and RdRP-ID37 showed no misamplification even after 120 min. Overall, using five primers -instead of six- obviously delayed the occurrence of misamplification. However, all other primer sets, except the E-ID1 and RdRP-ID37, still led to misamplification in the NTC tubes. This result verified the significant performance of E-ID1 primer set in the fluorometric and colorimetric RT-LAMP assays.

## Discussion

Diagnostic tests are the first line of defense against the spread of pandemics. Such tests must be accessible, fast, and simple with high diagnostic accuracy to be used for POC in areas where resources are limited. Alongside the gold standard RT-PCR, alternative molecular and serological techniques are being widely explored to develop rapid, accurate and cost-effective tests to efficiently diagnose COVID-19^8^. The simplicity of colorimetric RT-LAMP technique allows it to be used as a POC testing alternative for large-scale screening. In this work, two RT-LAMP assays (colorimetric and fluorometric) targeting the *E* gene of SARS-CoV-2 were developed. The colorimetric RT-LAMP has the advantage of simple and cost-effective reaction conditions in addition to visual interpretation of the results^16^. Its diagnostic accuracy, however, must be comparable to the gold standard RT-PCR to consider this technique reliable to avoid false results and their undesired consequences. In the colorimetric detection, 94.5% of the results were consistent with RT-PCR (Table S4). However, nine samples contradicted the RT-PCR results, so they were considered inconclusive. Also, the reaction was monitored for 120 min to determine the minimum time required for color change. It was observed that the color in high viral load samples (15 < Ct < 25) started to change after ~ 20 min, and lower viral load samples (35 ≤ Ct ≤ 40) began to change to yellow after 30–40 min (Fig. 6b). Over 50% of positive samples changed to yellow at 30 min (Fig. 6c). In addition, the difference in the ΔOD readings between positive and negative samples was statistically significant (*p* < 0.0001, 95% CI) (Fig. 6a). Overall, the sensitivity of the colorimetric RT-LAMP reaction greatly depends on the assay’s LoD, sample quality, viral load, and time of sample collection from the disease onset^18^.

The fluorometric RT-LAMP assay also showed a great performance when testing the clinical samples, where 96.7% of the results agreed with RT-PCR. The fluorometric and colorimetric results agree in all tested samples except the inconclusive ones, namely J20 and J43 (Figure S2). These inconclusive results can be attributed to the low viral load in these samples (Ct > 30). They can also be caused by the insufficient pH drop required for its yellow color transformation. The pH of the reaction solution is also affected by the elution buffer used during the sample’s RNA extraction process^11^. One clinical specimen (J15) was detected in both colorimetric and fluorometric RT-LAMP but was negative in RT-PCR (Table S3). Accordingly, both assays had higher sensitivity than RT-PCR in detecting SARS-CoV-2 in this specimen.

Even though RT-LAMP assays can be designed to specifically discriminate a variant^25^ or ultimately paired with next-generation sequencing to enable differential diagnosis^26^, the purpose of our assay is to target conserved, non-mutated regions of the most recent VOC, but are uncommon for other SARS to be used as a cost-effective POC diagnostic tool. So, this assay is expected to detect any SARS-CoV-2 variant, including the heavily mutated Omicron. Indeed, even with Omicron-specific point mutation (C/T) at the FIP binding site of the E-ID1 set (Figure S3), this primer set efficiently detected both the clinical and synthetic (Twist Bioscience) Omicron variant (Figs. 4, 7). The five primers in this set (E-ID1) caused a delayed detection time (~ 50 min) in positive specimens compared to other primer sets composed of six primers (~ 30–40 min), as demonstrated in Fig. 1. However, other primer sets (N-ID15, N-ID15n1L, S-ID17, S-ID24, and RdRp-ID37) had non-specific amplification in NTC appeared in 40 min (N-ID15 and S-ID17). The misamplification was apparent in all tested primer sets within 50–60 min, except the E-ID1. Regardless of the late color development, the E-ID1 primer set showed high stability at a longer reaction time when the incubation lasted up to 2 h (Fig. 7). The advantage of five primers in reducing misamplification is not limited to the E-ID1 primer set; as observed in Fig. 7. Other primer sets also showed better stability at longer reaction times. For instance, set N-ID15n1L with six primers showed misamplification in NTC after 50 min (Fig. 1), while the stability lasted up to 120 min using five primers (Fig. 7; Table S2). Additionally, the set RdRp-ID37 showed no misamplification with five primers (Fig. 7) but did so after 50 min when six primers were used in this set (Fig. 1). As expected, the time-to-detection is longer when excluding the LF primer in each set; however, the goal here is to increase LAMP primers’ stability to detect low viral load samples without false positive results. Higher primer stability enables optimizing the reaction conditions by adding components or varying the concentrations of existing components to enhance the detection speed without risking non-specific amplifications. For this reason, we tested tripling the Bst 2.0 DNA polymerase concentration to compensate for the reduction in the number of LAMP primers (from six to five). Increasing the enzyme concentration up to three times typically causes non-specific amplification in NTC due to the number of primers and high polymerase activity; however, this was not seen with E-ID1. This increase in the enzyme concentration would also increase the assay cost; nonetheless, the developed assays are expected to be more accurate in eliminating false-positive results.

A study by Jamwal et al.^27^ designed the same E-ID1 primer set/sequences and studied its performance on fluorometric RT-LAMP, which is expected because of the small size of the targeted *E* gene (228 bases) and the same primer design program was used. However, this is the first study to test this primer set using the colorimetric and fluorometric RT-LAMP methods. Also, the performance of this primer set was improved by optimizing the enzyme concentration and adding reaction enhancers like GuHCl. Indeed, after optimization, the detection time of the colorimetric assay was over 10 min earlier than pre-optimization when testing the same positive samples (Fig. 3). It also detected the clinical samples (i.e., J13, J31, J41, and J44) previously undetected (before optimization), confirming its effectiveness (Table S1). We tested the effect of Bst 3.0 DNA polymerase compared to the widely used Bst 2.0 DNA polymerase to choose the enzyme that better matches the primers (Figure S1). Our results indicate that Bst 2.0 was faster than Bst 3.0 DNA polymerase in detecting positive sample triplicates (Figure S1), concluding that this assay has a faster and more efficient detection when Bst 2.0 enzyme is used. Furthermore, the addition of GuHCl to the RT-LAMP reaction has enhanced the detection time and sensitivity. For instance, Zhang et al.^24^ reported up to a ten-fold increase in sensitivity and speed in low viral RNA samples; this addition did not increase misamplification in NTC. It is hypothesized that GuHCl enhances base pairing between primers and their targets, significantly improving detection speed and sensitivity^24^. Another study by Dudley et al. revealed that addition of GuHCl had lowered the LoD^22^. The improvement was evident with final concentrations between 40 and 60 mM in a 25 µL reaction volume, with 40 mM being the optimum recommended concentration https://international.neb.com^24^. Most studies reported this enhancement from GuHCl on RNA-extracted clinical samples but not in direct ones^22,23^. Based on that, we used 40 mM GuHCl in the colorimetric and fluorometric RT-LAMP reactions; both resulting in 3–7-min earlier amplifications in positive reactions with GuHCl. Adding GuHCl enhanced the detection time in this reaction. Still, it did not improve the sensitivity in detecting the inconclusive sample J43 (positive in RT-PCR and fluorometric RT-LAMP but negative in colorimetric RT-LAMP). In general, the effect of GuHCl varies depending on the primers used in the reaction. After optimizing the reaction conditions, our assay detects down to 20 copies/µL, or 500 copies/reaction. A study designed an *E* gene primer set composed of four primers with a detection limit of 2000 copies/reaction, which signifies the role of loop primer in improving sensitivity^28^. Our LoD is comparable to the one obtained by Zhang et al.^29^, who designed primer sets that detected down to 480 copies/reaction. Also, another study detected lower copy numbers (10 copies/µL) using primers that target the *ORF1ab* gene^30^. The LoD depends on the assays’ reaction conditions and the primers’ binding sites. However, none of these studies focused specifically on eliminating misamplifications in NTC that can appear during the extended reaction time.

Generally, the reaction conditions of our developed colorimetric RT-LAMP assay agree with the previous studies that used the same reagents from WarmStart (NEB, USA), being 30–60 min incubation time at 65 °C^11,13,14,18,19,25,31–36^. In the fluorometric RT-LAMP assay, we used LavaLAMP RNA enzyme (Lucigen, Middleton, WI, USA), which has an activation temperature of 68–74 °C. Accordingly, based on primers’ melting temperature (Tm) and after optimizing reaction conditions, 40–60 min at 70 °C were the optimum settings. Raddaz et al.^37^ also optimized their RT-LAMP assay by testing varying concentrations of the reaction components, such as Bst DNA polymerase, primers, and MgSO_4_ concentrations, which significantly enhanced the sensitivity up to ten folds compared to the WarmStart protocol without optimization. Unlike our study, GuHCl addition had no effect in improving time to reaction (TTR) response in their assay since it was a duplex (targeting both *N* and *ORF1ab* genes), which supports the idea that the effect of GuHCl varies depending on the primers used. In addition, they observed a higher misamplification rate with increased Bst concentration^36^. Not many studies addressed a solution to misamplifications in NTC or negative samples. Only a few studies suggested not exceeding the reaction time to over 30–60 min or using quenched fluorescent primers^36^, highlighting the advantage of our assay in eliminating this issue despite increasing the concentration of Bst DNA polymerase.

Both colorimetric and fluorometric assays showed excellent performance in diagnosing COVID-19, being highly selective against SARS-CoV-2 variants since the primers showed no cross-reactivity against other respiratory viruses in-silico and in-vitro. The primer set E-ID1 was only used in one study to diagnose SARS-CoV-2 using densitometry and agarose gel electrophoresis to detect RT-LAMP amplification products. However, this primer set did not perform well in their research, so they did not test it further^27^. In addition, their study used Bst 3.0 enzyme to show the fastest amplification among the other tested enzymes, while our primers performed best when Bst 2.0 enzyme was used. Hence, our optimization to enhance the performance of E-ID1 in the colorimetric and fluorometric RT-LAMP assay is novel. The assays can be improved by testing alternative enzymes with high strand displacement activity or developing homemade reagents to reduce cost. Also, the colorimetric RT-LAMP assay can be developed for self-testing at home by freeze-drying or lyophilizing the reagents, as achieved by Song et al.^38^, who found that the lyophilized RT-LAMP reactions have less false-positive results compared to solution-based reagents. Lyophilization extends the reagent’s shelf life, allows storing at room-temperature, and overcomes cold-shipping costs. We found that this assay successfully detected SARS-CoV-2 starting from 20 min. The reaction can be extended up to 120 min –without misamplification– particularly for patients with low viral loads. In that regard, the current RT-LAMP test recommends the use of five primers that are rapid and sensitive in detecting COVID-19 infection.

## Conclusion

This study aims to improve one of the limiting factors of the RT-LAMP technique, which is the non-specific amplifications leading to false-positive results. In general, false-positive results occur within 30 min, therefore, current protocols do not recommend exceeding the reaction time over 30 min. This leads to difficulties in diagnosing particularly low viral load samples. To eliminate the misamplifications, we designed a primer set, namely E-ID1, consisting of five primers that exclude the loop forward (LF) primer. Results showed that the five-primer design provides stable primer targeting and avoids misamplification for 120 min. Optimizing the colorimetric and fluorometric RT-LAMP assays decreased the detection time. The colorimetric technique uses only a heating block, without any bulky or expensive instruments. The test results can be visually interpreted through a simple color change. The primers used in this study target a conserved region in the SARS-CoV-2 *E* gene regardless of the variant. Both colorimetric and fluorometric assays performed remarkably, with sensitivities of 89.5% and 92.2%, respectively, making them successful candidates for replacing the gold standard RT-PCR, thereby greatly contributing to improving the economy. Future work would include improving the accuracy and cost of the assays by further optimization or using homemade reagents. Also, the colorimetric RT-LAMP assay has the potential to be developed for in-home use for self-diagnosis by lyophilizing the reagents. The colorimetric RT-LAMP technique reduces time and load on healthcare workers, making it suitable for POC testing in local screening centers, emergency departments, and airports.

## Methods

### Alignment of SARS-CoV-2 genome sequences and RT-LAMP primer design

SARS-CoV-2 genome sequences were downloaded from the GISAID (Global Initiative on Sharing All Influenza Data, https://www.gisaid.org) and NCBI GenBank https://www.ncbi.nlm.nih.gov/genbank/ databases. At the time of writing, 14.8 million hCoV-19 genome sequences were submitted worldwide. The downloaded genome sequence information was sampled from different continents and included variants such as Alpha (B.1.1.7, Q.1-Q.8), Beta (B.1.351, B.1.351.2, B.1.351.3), Gamma (P.1, P.1.1, P.1.2), Delta (B.1.617.2), and Omicron (B.1.1.529) to identify the most conserved regions for RT-LAMP primer design. This way, the designed assay would target the viral gene as sensitive, specific, and accurate as possible, regardless of the variant. Also, whole genome sequences of other SARS-CoVs (AY278491.2, AY502924.1, AY502927.1, AY559094.1, AY613947.1, and NC_004718.3) were downloaded from the NCBI database. Then, a comparative analysis was made by aligning the full genome sequences at the base level with Clustal Omega program https://www.ebi.ac.uk/Tools/msa/clustalo/. Afterward, the viral target regions were selected in conformity with COVID-19 testing directives of the CDC, WHO, and EU Commission. The mutation sites were identified using JalView (v2.11.1.3) program^39^. The conserved regions specific for SARS-CoV-2 but not for the other SARS-COV species were selected for the primers binding sites. All RT-LAMP primer sets, each containing five to six primers, were designed using the PrimerExplorer V5 program https://primerexplorer.jp/e that target the conserved region in the *N*, *S*, *RdRp*, and *E* genes. In addition, the OligoAnalyzer tool from Integrated DNA Technologies (IDT) https://eu.idtdna.com/pages/tools/oligoanalyzer was used to check that each primer set would not form unwanted secondary structures like homodimer, hetero-dimer, and hairpins. The oligonucleotides were synthesized, lyophilized and desalted (Alpha DNA, Montreal, Canada) http://www.alphadna.com. All the designed primer sets were tested on SARS-CoV-2 positive control (PC)—a mixture of verified high SARS-CoV-2-loaded specimens—, synthetic SARS-CoV-2 RNA (Twist synthetic RNA control 51 (EPI_ISL_7718520), Twist Bioscience, USA), and non-template control (NTC)—distilled water—. The primer sets that showed the best performance were chosen for further testing and analysis. In a reaction volume (25 µL), the primer concentrations (10×) were settled as follows: 2 µM for F3 and B3, 4 µM for LF and LB, and 16 µM for FIP and BIP. Sequences of all primer sets are shown in Table 1.

**Table 1:** Sequences of each RT-LAMP primer set used in this study.

Primer set	Sequence (5′–3′)
N-ID15	F3: AGATCACATTGGCACCCG B3: CCATTGCCAGCCATTCTAGC FIP: TGCTCCCTTCTGCGTAGAAGCCAATGCTGCAATCGTGCTAC BIP: GGCGGCAGTCAAGCCTCTTCCCTACTGCTGCCTGGAGTT LF: GGCAATGTTGTTCCTTGAGGAAGTT LB: CACGTAGTCGCAACAGTTCAA
N-ID15n1L*	F3: AGATCACATTGGCACCCG B3: CCATTGCCAGCCATTCTAGC FIP: TGCTCCCTTCTGCGTAGAAGCCAATGCTGCAATCGTGCTAC BIP: GGCGGCAGTCAAGCCTCTTCCCTACTGCTGCCTGGAGTT LF: GCAATGTTGTTCCTTGAGGAAGTT LB: GTTCCTCATCACGTAGTCGCAACA
S-ID17	F3: TCTTTCACACGTGGTGTT B3: GTACCAAAAATCCAGCCTC FIP: CATGGAACCAAGTAACATTGGAAAACCTGACAAAGTTTTCAGATCC BIP: CTCTGGGACCAATGGTACTAAGAGGACTTCTCAGTGGAAGCA LF: GGTAAGAACAAGTCCTGAGTTGAA LB: GTTTGATAACCCTGTCCTACCATT
S-ID24	F3: GGTGTTTATTACCCTGACAAAG B3: GTACCAAAAATCCAGCCTC FIP: CATGGAACCAAGTAACATTGGAAAATTTTCAGATCCTCAGTTTTACATTC BIP: CTCTGGGACCAATGGTACTAAGAGGACTTCTCAGTGGAAGCA LF: GAAAGGTAAGAACAAGTCCTGAGT LB: GTTTGATAACCCTGTCCTACCATT
E-ID1*	F3: TCATTCGTTTCGGAAGAGA B3: AGGAACTCTAGAAGAATTCAGAT FIP: TGTAACTAGCAAGAATACCACGAAACAGGTACGTTAATAGTTAATAGCG BIP: GCTTCGATTGTGTGCGTACTCGAGAGTAAACGTAAAAAGAAGG LB: GCTGCAATATTGTTAACGTGAGTC
RdRp-ID37	F3: ACAAAGCCTTACATTAAGTGG B3: CACCATCAACAAATATTTTTCTCAC FIP: TGGGTGGTATGTCTGATCCCAATAGATTTGTTAAAATATGACTTCACGG BIP: TGTGTTAACTGTTTGGATGACAGATTGTAAGTGGGAACACTGT LF: ACGGTCAAAGAGTTTTAACCTCTCT LB: GCATTCTGCATTGTGCAAACT

F3, forward outer; FIP, forward inner; LF, loop forward; B3, backward outer; BIP, backward inner; LB, loop backward. All the primers were designed by using the PrimerExplorer program. *Jamwal et al.^27^ designed and used these primer sets.

### Fluorometric RT-LAMP reaction

The fluorometric RT-LAMP reactions were carried out in 25 μL volume. Two brands were tested in the fluorometric detection to choose the most compatible reagents that show the best performance: WarmStart LAMP kit (DNA and RNA) (New England Biolabs (NEB), USA) and LavaLAMP RNA component kit (Lucigen, Middleton, WI, USA). Unless otherwise stated, a LavaLAMP master mix contained 2.5 µL 10× LavaLAMP RNA buffer, 1.25 µL 100 mM MgSO_4_, 2 µL 10 mM dNTP solution, 1 µL green fluorescent dye (20×), 1 µL LavaLAMP RNA enzyme (Lucigen, USA), 2.5 µL primer mixture (10×), 5 µL template (or dH_2_O for NTC), and dH_2_O up to 25 µL. In the fluorometric assay using WarmStart kit, the master mix included 2.5 µL WarmStart isothermal amplification buffer (10×), 1.5 µL 100 mM MgSO_4_, 3.5 µL 10 mM deoxynucleotide (dNTP) solution, 0.5 µL LAMP fluorescent dye (50×), 8000 U/mL Bst 2.0 WarmStart DNA polymerase, 0.5 µL WarmStart RTx reverse transcriptase (NEB, USA), 2.5 µL primer mix (10×), 2 µL template (or dH_2_O for NTC), and dH_2_O up to 25µL. Reactions were performed in a 7500 Fast Real-Time PCR System (Thermo Fisher Scientific, Waltham, MA, USA) https://www.thermofisher.com, at 65 °C for Warmstart and 70 °C for LavaLAMP. The reactions were settled for 80 cycles of 45 s (unless otherwise stated) using FAM filter as the reporter dye channel. The normalizing reporter (Rn) is the intensity of the reporter dye’s fluorescence emission divided by the intensity of the passive reference dye’s fluorescence emission. ΔRn unit on the y-axis signifies the fluorescent signal magnitude determined by (Rn+) − (Rn−), where Rn+ is the Rn value of all reaction components and Rn− is the Rn value of unreacted components.

### Colorimetric RT-LAMP detection

For a 25 µL total reaction volume, unless otherwise specified, the colorimetric RT-LAMP mixture is composed of 12.5 µL WarmStart Colorimetric LAMP 2× master mix with UDG (NEB, USA), 2.5 µL 10× primer mix, 2 µL template (or dH_2_O for NTC), and dH_2_O up to 25 µL. The mixture is incubated in a water bath (Thermo Fisher Scientific, Waltham, MA, USA) https://www.thermofisher.com, set at 65 °C for 60 min or until a color change appears.

### Agarose gel electrophoresis

Positive or negative amplifications upon RT-LAMP reactions were validated by loading RT-LAMP products in 2% agarose gel prepared with VisualaNA (A) DNA Stain from Molequle-On (Auckland, New Zealand) http://molequle-on.com and run in electrophoresis unit (Analytik Jena) for 45 min operating at 100 V. Then, the gel is visualized under a UV-trans illuminator (ChemiDoc™ XRS + System with Image Lab™ Software, Bio-Rad, USA). Successful amplifications in positive samples were visualized as ladder-type DNA bands.

### Collection of samples and validation of results

Multiple validations were conducted on real clinical specimens in collaboration with Johns Hopkins Aramco Healthcare (JHAH), authorized to store and analyze SARS-CoV-2 samples from patients, which were leftover RNA-extracted samples provided by JHAH. A sufficient number of samples (165 validated Delta and Omicron SARS-CoV-2 positive or negative RNA samples) were simultaneously tested and validated with the herein-developed colorimetric and fluorometric RT-LAMP assays, and other commercially or in-house developed RT-qPCR kits^40,41^. The tests were performed in the genetics lab of the Department of Genetic Research (Institute for Research and Medical Consultations (IRMC), Imam Abdulrahman bin Faisal University, Dammam, KSA). The limit of detection (LoD) was determined using serial dilutions of synthetic SARS-CoV-2 RNA control (Twist synthetic RNA control 51 (EPI_ISL_7718520), Twist Bioscience, USA). The specificity was determined by testing other respiratory virus RNA (parainfluenza virus 3, enterovirus, rhinovirus, human metapneumovirus A + B, parainfluenza virus 4, bocavirus, and coronavirus 229 E), which were leftover clinical RNA samples provided from King Fahad Specialist Hospital (KFSH) (Dammam, KSA). Eventually, the following results for further validation were obtained: (1) RT-PCR results from the abovementioned hospitals, (2) RT-PCR results from in situ lab, (3) fluorescent RT-LAMP, (4) colorimetric RT-LAMP, and (5) agarose gel electrophoresis.

### RT-PCR assay

Since RT-PCR assay is accepted as the gold standard method, the positivity or negativity of the collected specimens was tested to verify the RT-LAMP results. RNA samples were used as a templates by targeting at least two viral genes (*RdRp*, *N*, and *E*) and a human *RP* gene as the internal control, as described earlier^40,41^. A 20 µL reaction mixture was composed of 2 µL of 10× Buffer (Procomcure Biotech, Austria), 0.25 µL of 10 mM dNTP (Procomcure Biotech, Austria), 0.2 µL of 1 U/µL uracil-DNA glycosylase (UDG) (NEB, USA), 0.25 µL molecular grade dimethyl sulfoxide (DMSO) (Sigma-Aldrich), primer and probe mixture (Molequle-On, New Zealand), 0.4 µL of 2 U/µL VitaTaq HS polymerase (Procomcure Biotech, Austria), 0.5 µL of 200 U/µL M-MuLV Reverse Transcriptase (NEB, USA), 5 µL RNA template and RNase/DNase-free ddH_2_O up to 20 µL. In addition, no-template control (NTC) was used to detect any possible misamplifications. The reactions were run in a real-time PCR instrument (Applied Biosystems™ real-time PCR 7500). The samples having a threshold cycle (Ct) score over 37 (Ct > 37) were considered negative. The SARS-CoV-2 positive specimens were identified if the Ct number was ≤ 37 with a sigmoidal amplification curve.

### Ethical approval

The study is approved by the Institutional Review Board (IRB) at Imam Abdulrahman bin Faisal University (IAU) with an IRB number of IRB-2020-13-406. All methods were carried out in accordance with relevant guidelines and regulations. The de-identified samples left over after the completion of diagnostic tests were used; hence this study requires no informed consent form as per institutional ethics committee regulations.

### Statistical analysis

The difference between the positive and negative samples’ color development was determined spectrophotometrically by the mean values of the optical density (ΔOD) for the colorimetric quantification. This was done by transferring equal amounts of five positive and five negative clinical samples in a 96-well cell culture plate (Thermofisher Scientific) and reading absorbance values at wavelengths 434 and 560 nm using a plate reader (BioTek Synergy HTX microplate reader, Agilent). The difference in optical densities was considered statistically significant if the *p* value < 0.05 in an unpaired *t *test performed using GraphPad Prism 9.0 (GraphPad Software, USA).

## Supplementary Information

Supplementary Information.

## Data Availability

The datasets generated and analyzed during the current study are available from the corresponding author upon reasonable request.
