# Supplementary material for: Development of loop-mediated isothermal amplification (LAMP) assays using five primers reduces the false-positive rate in COVID-19 diagnosis
Source: Sci Rep. 2023 Mar 28;13:5066. doi: 10.1038/s41598-023-31760-z (PMC10044074; doi:10.1038/s41598-023-31760-z)
Supplement: Supplementary file 1 — Supplementary Information. [file 41598_2023_31760_MOESM1_ESM.docx]

**SUPPLEMENTARY INFORMATION**

**Development of Loop-mediated Isothermal Amplification (LAMP) Assays Using Five Primers Reduces the False-positive Rate in COVID-19 Diagnosis**

**Galyah Alhamid^1,2^, Huseyin Tombuloglu^2^*, Ebtesam Alsuhaimi^3^**

^1^Master Program of Biotechnology, Institute for Research and Medical Consultations, Imam Abdulrahman Bin Faisal University, Dammam, Saudi Arabia

^2^Department of Genetics Research, Institute for Research and Medical Consultations (IRMC), Imam Abdulrahman Bin Faisal University, 31441, Dammam, Saudi Arabia.

^3^Biology Department, College of Science and Institute of Research and Medical Consultations (IRMC), Imam Abdulrahman Bin Faisal University, Dammam 31441, Saudi Arabia


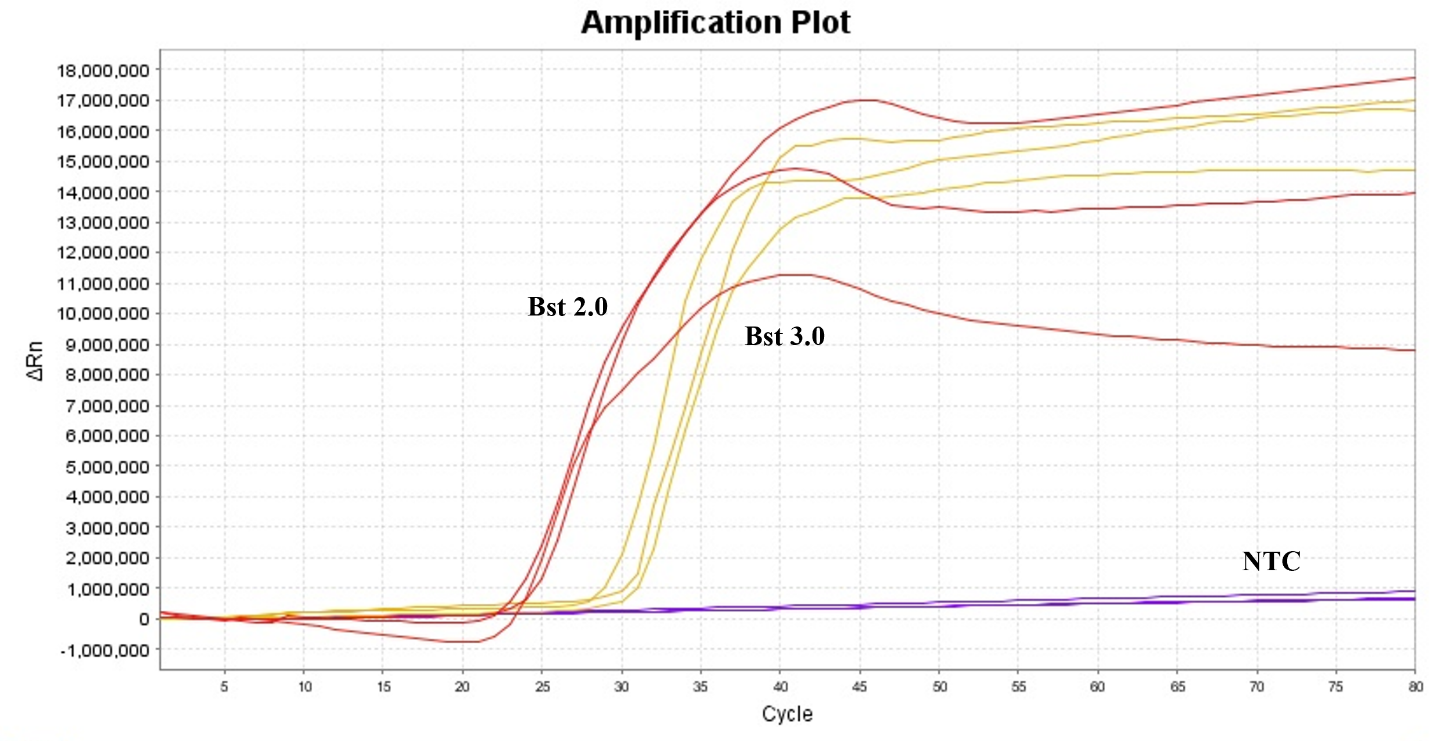


**Figure S1** Comparison between Bst 2.0 (red) and Bst 3.0 (yellow) DNA polymerase performance on three replicates of positive and negative controls (PC and NC) in the fluorometric RT-LAMP using WarmStart reagents. No improvement was evident in reactions using Bst 3.0 enzyme; thus, Bst 2.0 DNA polymerase has been used for further testing on E-ID1 primers.


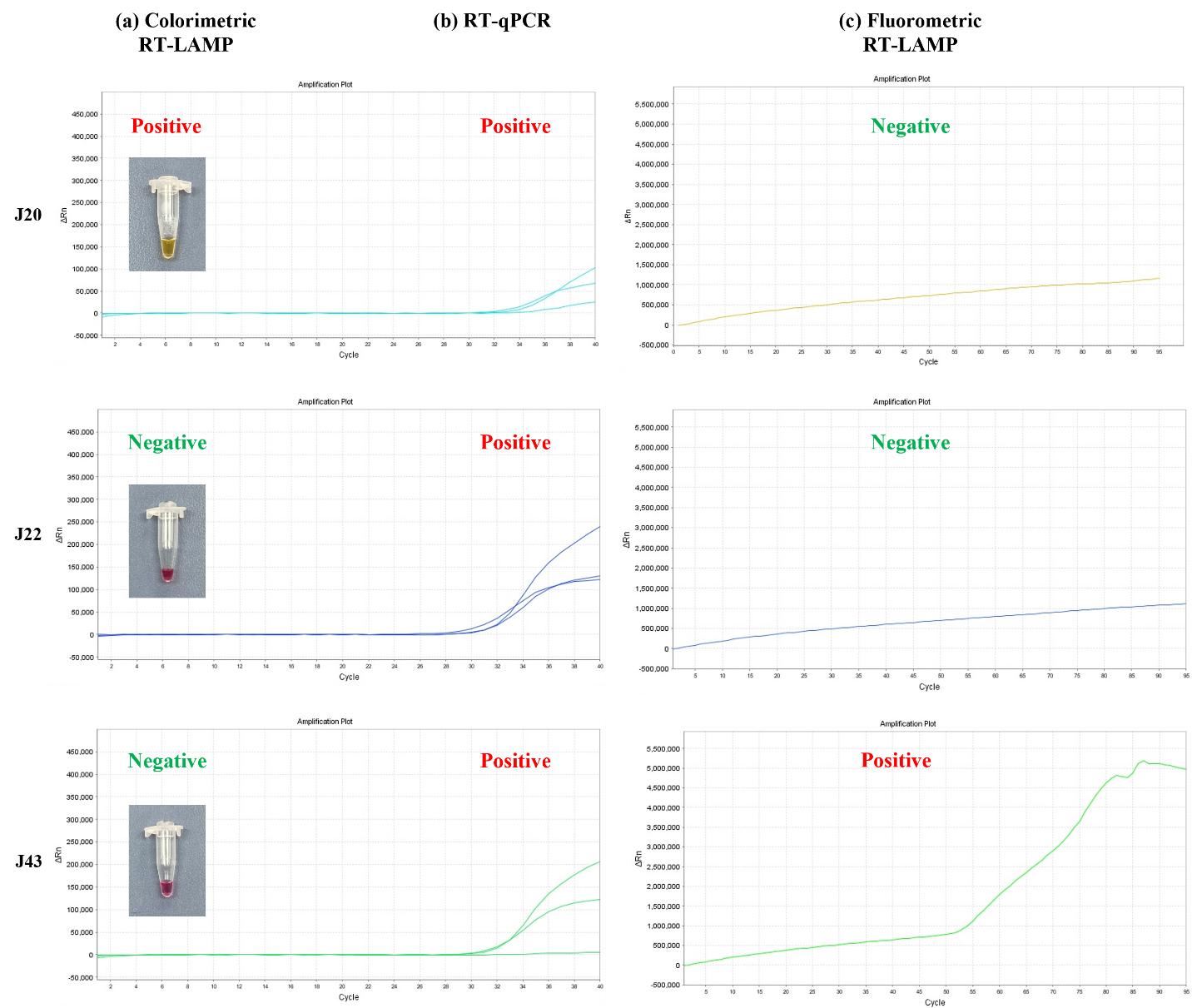


**Figure S2** Comparison of **(a)** colorimetric RT-LAMP, **(b)** RT-PCR, and **(c)** fluorometric RT-LAMP results of three inconclusive clinical specimens (J20, J22, and J43).


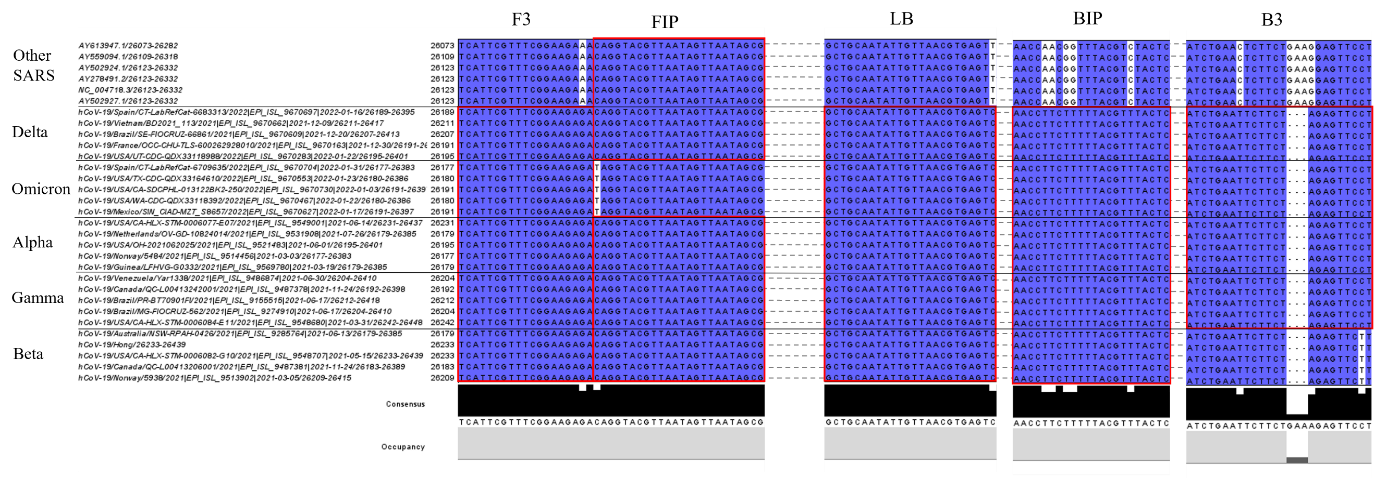


**Figure S3** E-ID1 primers binding sites on the *E* gene of SARS-CoV-2 variants and other SARS viruses. This primer sequences bind to a conserved region in SARS-CoV-2 but uncommon for other SARS. Only one point mutation exists at the forward inner primer (FIP) binding site.


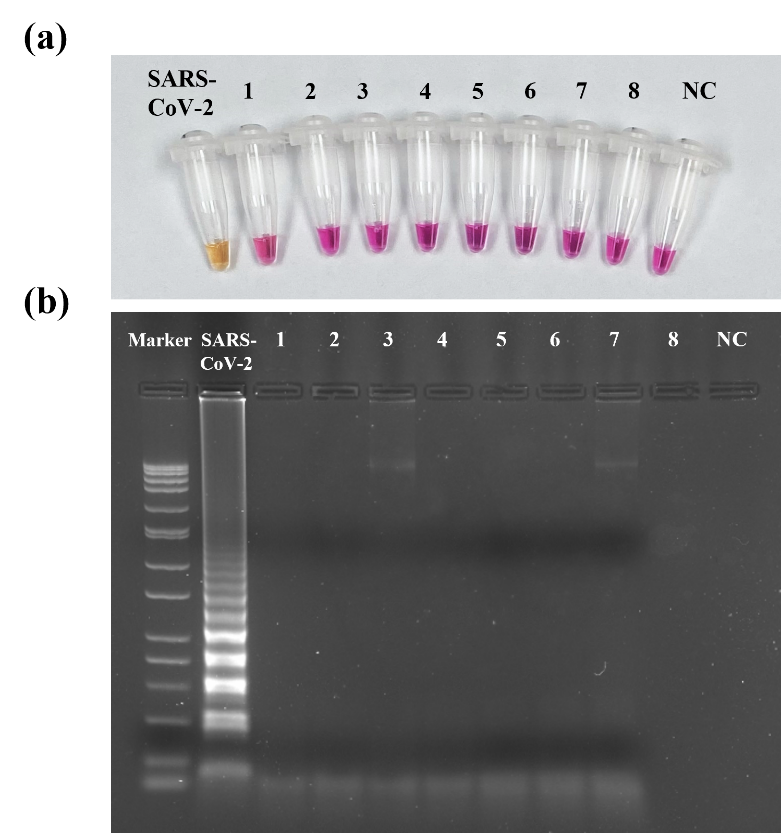


**Figure S4** **(a)** Selectivity of E gene primers colorimetrically tested against clinical samples infected with SARS-CoV-2 along with other respiratory viruses (1-8), these include coronavirus 229 E, parainfluenza virus 3, parainfluenza virus 4, human metapneumovirus A+B, bocavirus, enterovirus, and rhinovirus. The positive color change is seen in SARS-CoV-2 sample only. **(b)** Loading the samples in 2% agarose gel post-reaction showed a ladder-type pattern in COVID-19-infected sample only and nothing in samples with other respiratory viruses, indicating high specificity of the primers against SARS-CoV-2.


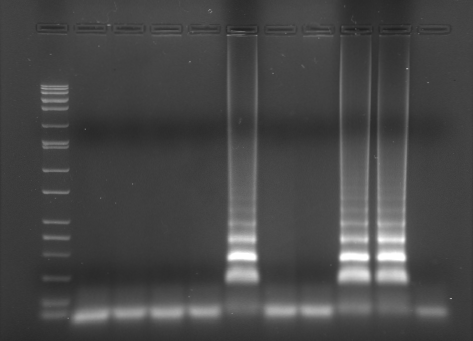


**Figure S5** The full gel image of Figure 4(b).


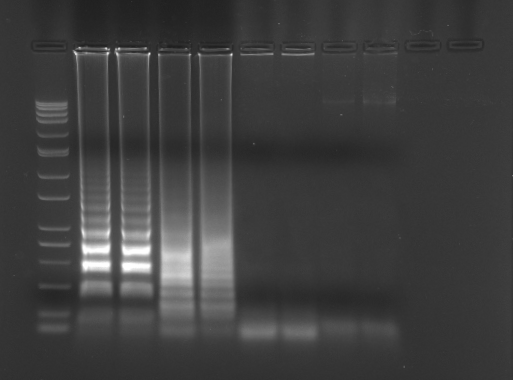


**Figure S6** The full gel image of Figure 5(c).

**
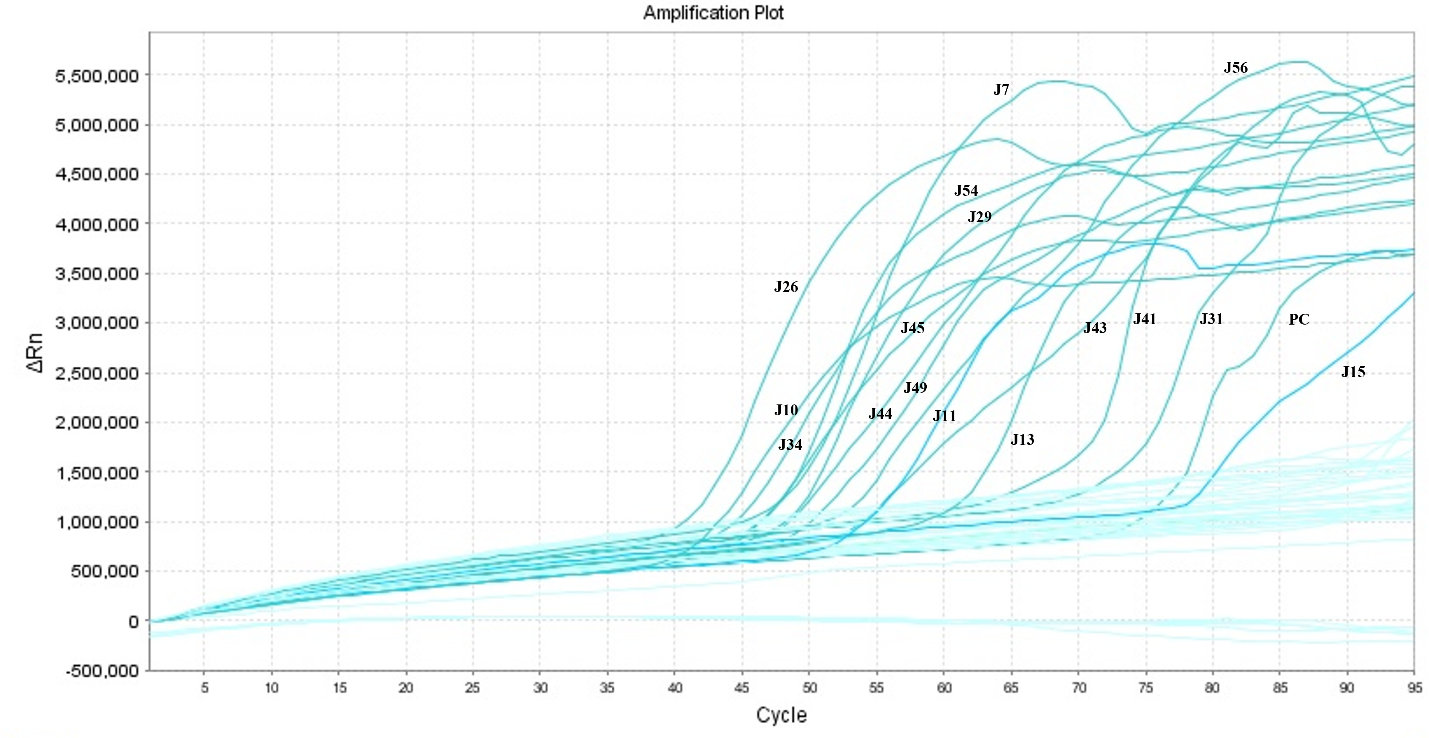
**

**Figure S7** The optimized fluorometric RT-LAMP assay tested on 50 clinical samples. 17 out of 50 were positive, indicated by the blue S-shaped amplification curves, and 33 out of 50 were negative, shown in light blue color (the negative samples in this run included J5, J6, J8, J9, J12, J14, J16, J17, J18, J19, J20, J21, J22, J23, J24, J25, J27, J28, J30, J32, J33, J35, J38, J39, J40, J42, J46, J47, J48, J50, J51, J52, and J53).

**Table S1** Summary of the detection time difference between the fluorometric RT-LAMP assays before and after optimization on positive clinical samples.

| **Samples** | **RT-PCR (Ct)** | **WarmStart**  **before optimization (minutes)** | **WarmStart**  **after optimization (minutes)** | **LavaLAMP™**  **before optimization (minutes)** | **LavaLAMP™**  **after optimization (minutes)** |
| --- | --- | --- | --- | --- | --- |
| PC mix | 19.72 |  | 20  (42 with 2x Bst 2.0) | 37 | 34 |
| P1 | 13.78 | 41 | 30 (2x Bst 2.0) | 35 | 26 |
| P13 | 10.27 | 42 | 25 (2x Bst 2.0) | 33 | 27 |
| P14 | 14.41 | 37 | 28 (2x Bst 2.0) | 33 | 27 |
| J13 | 24.01 | Negative | 42 (2x Bst 2.0) | Negative | 41 |
| J31 | 24.6 | Negative | 34 | 44 | 45 |
| J36 | 26.41 | Negative | Negative | Negative |  |
| J37 | 29.43 | Negative | Negative | Negative |  |
| J41 | 27.61 | Negative | 59 |  | 51 |
| J44 | 23.07 | Negative | 58 |  | 43 |

Abbreviations: RT-qPCR, reverse-transcription quantitative polymerase chain reaction; PC, positive control.

**Table S2** Comparison between using six and five primers of each primer set in producing misamplification in NTC (n = 5).

| **Primer set** | **Time-to-misamplification in NTC (in 120 minutes)** | |
| --- | --- | --- |
|  | **Six primers** | **Five primers** |
| **E-ID1** | **-** | None |
| **N-ID15** | 40 ± 2.1 | 95 ± 5.0 |
| **N-ID15n1L** | 50 ± 1.8 | 120 ± 1.6 |
| **S-ID17** | 40 ± 3.0 | 90 ± 2.5 |
| **S-ID24** | 60 ± 1.2 | 80 ± 4.1 |
| **RdRp-ID37** | 50 ± 2.0 | None |

Abbreviations: NTC, no template control.

**Table S3** RT-PCR, colorimetric, and fluorometric RT-LAMP results of the tested clinical samples

| **Sample ID** | **In-house RT-PCR (Ct)** | | | | **RT-PCR (JHAH)** | **Colorimetric RT-LAMP** | **Fluorometric RT-LAMP** |
| --- | --- | --- | --- | --- | --- | --- | --- |
|  | ***N*** | ***RdRp*** | ***RP*** | **Result** |  |  |  |
| P1 | 13.78 | 19.50 | 27.47 | + | + | + | + |
| P13 | 10.27 | 11.50 | 24.79 | + | + | + | + |
| P14 | 14.41 | 17.40 | 27.49 | + | + | + | + |
| P15 | 34.35 | 35.10 | 26.55 | + | + | - | - |
| J5 | 34.15 | 38.45 | 30.79 | + | + | - | - |
| J6 |  |  |  | - | - | - | - |
| J7 | 17.01 | 16.50 | 27.18 | + | + | + | + |
| J8 |  |  |  | - | - | - | - |
| J9 |  |  |  | - | - | - | - |
| J10 | 13.97 | 15.40 | 27.57 | + | + | + | + |
| J11 | 14.41 | 13.20 | 27.49 | + | + | + | + |
| J12 |  |  |  | - | - | - | - |
| J13 | 24.01 | 24.83 | 27.66 | + | + | + | + |
| J14 |  |  |  | - | - | - | - |
| J15 |  |  |  | - | - | + | + |
| J16 |  |  |  | - | - | - | - |
| J17 |  |  |  | - | - | - | - |
| J18 |  |  |  | - | - | - | - |
| J19 |  |  |  | - | - | - | - |
| J20 | 31.20 | 32.56 | 32.45 | + | + | + | - |
| J21 |  |  |  | - | - | - | - |
| J22 | 29.09 | 29.42 | 25.05 | + | + | - | - |
| J23 |  |  |  | - | - | - | - |
| J24 |  |  |  | - | - | - | - |
| J25 |  |  |  | - | - | - | - |
| J26 | 20.24 | 23.20 | 25.25 | + | + | + | + |
| J27 |  |  |  | - | - | - | - |
| J28 |  |  |  | - | - | - | - |
| J29 | 24.35 | 35.40 | 29.81 | + | + | + | + |
| J30 |  |  |  | - | - | - | - |
| J31 | 24.60 | 24.33 | 28.45 | + | + | + | + |
| J32 |  |  |  | - | - | - | - |
| J33 |  |  |  | - | - | - | - |
| J34 | 17.76 | 16.50 | 27.28 | + | + | + | + |
| J35 |  |  |  | - | - | - | - |
| J36 | 26.41 | 26.46 | 32.14 | + | + | - |  |
| J37 | 27.43 | 28.49 | 30.75 | + | + | - |  |
| J38 |  |  |  | - | - | - | - |
| J39 |  |  |  | - | - | - | - |
| J40 |  |  |  | - | - | - | - |
| J41 | 27.61 | 18.76 | 19.46 | + | + | + | + |
| J42 |  |  |  | - | - | - | - |
| J43 | 29.73 | 29.98 | 33.84 | + | + | - | + |
| J44 | 23.07 | 23.84 | 31.26 | + | + | + | + |
| J45 | 18.96 | 28.69 | 32.27 | + | + | + | + |
| J46 |  |  |  | - | - | - | - |
| J47 |  |  |  | - | - | - | - |
| J48 |  |  |  | - | - | - | - |
| J49 | 27.49 | 27.29 | 25.71 | + | + | + | + |
| J50 |  |  |  | - | - | - | - |
| J51 |  |  |  | - | - | - | - |
| J52 |  |  |  | - | - | - | - |
| J53 |  |  |  | - | - | - | - |
| J54 | 28.99 | 30.20 | 29.24 | + | + | + | + |
| J55 |  |  |  | - | - | - | - |
| J56 | 31.54 | 32.96 | 29.98 | + | + | + | + |
| J57 |  |  |  | - | - | - | - |
| J58 | 32.36 | 31.85 | 32.65 | + | + | + | + |
| J59 |  |  |  | - | - | - | - |
| J60 |  |  |  | - | - | - | - |
| J61 |  |  |  | - | - | - | - |
| J62 |  |  |  | - | - | - | - |
| J63 |  |  |  | - | - | - | - |
| J64 |  |  |  | - | - | - | - |
| J65 | 22.80 | 26.70 | 28.48 | + | + | + | + |
| J66 | 27.95 | 27.94 | 26.49 | + | + | + | + |
| J67 |  |  |  | - | - | - | - |
| J68 |  |  |  | - | - | - | - |
| J69 |  |  |  | - | - | - | - |
| J70 |  |  |  | - | - | - | - |
| J71 | 31.60 | 32.41 | 24.50 | + | + | + | + |
| J72 | 33.46 | 33.89 | 31.83 | + | + | + | + |
| J73 |  |  |  | - | - | - | - |
| J74 | 26.33 | 32.07 | 24.31 | + | + | + | + |
| J75 |  |  |  | - | - | - | - |
| J76 |  |  |  | - | - | - | - |
| J77 |  |  |  | - | - | + | - |
| J78 |  |  |  | - | - | - | - |
| J79 |  |  |  | - | - | - | - |
| J80 |  |  |  | - | - | + | - |
| J81 | ND | 24.70 | 28.90 | + | + | + | + |
| J82 |  |  |  | - | - | - | - |
| J83 | 28.96 | 29.39 | 26.94 | + | + | + | + |
| J84 |  |  |  | - | - | - | - |
| J85 |  |  |  | - | - | - | - |
| J86 |  |  |  | - | - | - | - |
| J87 |  |  |  | - | - | - | - |
| J88 |  |  |  | - | - | - | - |
| J89 |  |  |  | - | - | - | - |
| J90 |  |  |  | - | - | - | - |
| J91 |  |  |  | - | - | - | - |
| J92 | 30.55 | 29.59 | 26.05 | + | + | + | + |
| J93 |  |  |  | - | - | - | - |
| J94 |  |  |  | - | - | - | - |
| J95 | 19.72 | 21.47 | 32.71 | + | + | + | + |
| J96 |  |  |  | - | - | - | - |
| J97 | 34.23 | ND | 27.08 | + | + | + | + |
| J98 |  |  |  | + | + | + | + |
| J99 |  |  |  | - | - | - | - |
| J100 |  |  |  | - | - | - | - |
| J101 |  |  |  | - | - | - | - |
| J102 | 34.40 | 32.33 | 32.36 | + | + | + | + |
| J103 |  |  |  | - | - | - | - |
| J104 | ND | 23.50 | 25.06 | + | + | + | + |
| J105 | 18.50 | 20.84 | 26.72 | + | + | + | + |
| J106 |  |  |  | - | - | - | - |
| J107 |  |  |  | - | - | - | - |
| J108 |  |  |  | - | - | - | - |
| J109 |  |  |  | - | - | - | - |
| J110 | ND | 33.92 | 31.50 | + | + | + | + |
| J111 |  |  |  | - | - | - | - |
| J112 | 17.18 | 19.02 | 29.43 | + | + | + | + |
| J113 |  |  |  | - | - | - | - |
| J114 |  |  |  | - | - | - | - |
| J115 | 38.43 | 32.21 | 29.41 | + | + | + | + |
| J116 | 38.94 | 35.40 | 36.45 | + | + | - | + |
| J117 |  |  |  | - | - | - | - |
| J118 |  |  |  | - | - | - | - |
| J119 |  |  |  | - | - | - | - |
| J120 |  |  |  | - | - | - | - |
| J121 | 32.27 | 29.99 | 34.30 | + | + | + | + |
| J122 |  |  |  | - | - | - | - |
| J123 |  |  |  | - | - | - | - |
| J124 | 32.27 | 29.96 | 22.65 | + | + | + | + |
| J125 |  |  |  | - | - | - | - |
| J126 |  |  |  | - | - | - | - |
| J127 |  |  |  | - | - | - | - |
| J128 |  |  |  | - | - | - | - |
| J129 |  |  |  | - | - | - | - |
| J130 |  |  |  | - | - | - | - |
| J131 | 13.06 | 15.17 | 31.03 | + | + | + | + |
| J132 |  |  |  | - | - | - | - |
| J133 | 15.55 | 18.96 | 29.39 | + | + | + | + |
| J134 |  |  |  | - | - | - | - |
| J135 |  |  |  | - | - | - | - |
| J136 |  |  |  | - | - | - | - |
| J137 | 38.57 | 36.02 | 29.02 | + | + | + | + |
| J138 |  |  |  | - | - | - | - |
| J139 |  |  |  | - | - | - | - |
| J140 |  |  |  | + | + | + | + |
| J141 |  |  |  | - | - | - | - |
| J142 |  |  |  | + | + | + | + |
| J143 |  |  |  | - | - | - | - |
| J144 |  |  |  | - | - | - | - |
| J145 | 18.67 | ND | 24.44 | + | + | + | + |
| J146 |  |  |  | - | - | - | - |
| J147 | 37.50 | 36.96 | 29.83 | + | + | + | + |
| J148 |  |  |  | + | + | + | + |
| J149 |  |  |  | - | - | - | - |
| J150 |  |  |  | - | - | - | - |
| J151 |  |  |  | - | - | - | - |
| J152 |  |  |  | - | - | - | - |
| J153 |  |  |  | + | + | + |  |
| J154 |  |  |  | - | - | - |  |
| J155 |  |  |  | + | + | + |  |
| J156 |  |  |  | + | + | + |  |
| J157 |  |  |  | - | - | - |  |
| J158 |  |  |  | - | - | - |  |
| J159 |  |  |  | - | - | - |  |
| J160 |  |  |  | - | - | - |  |
| J161 |  |  |  | + | + | + |  |
| J162 |  |  |  | - | - | - |  |
| J163 |  |  |  | - | - | - |  |
| J164 |  |  |  | - | - | - |  |
| J165 |  |  |  | - | - | - |  |
| PC mix | 19.72 | 21.47 | 32.71 | + | + | + | + |

ND: not determined.

JHAH: John Hopkins Aramco Healthcare

To evaluate the reliability of the diagnostic test, the below confusion matrices were used. Table S4 demonstrates the number of true positive (TP), true negative (TN), false positive (FP), and false negative (FN) values of 165 and 150 tested samples in the colorimetric and fluorometric assays, respectively. The gold standard RT-PCR was used as the reference to determine the TP and TN results among all tested samples. Accordingly, the assays’ sensitivity, specificity, accuracy, positive predictive value (PPV), and negative predictive value (NPV) were determined based on the following formulas:

**Table S4** True positive (TP), false positive (FP), true negative (TN) and false negative (FN) terms utilized to evaluate the diagnostic accuracy of the RT-LAMP method.

| Comparative method | Test method  **+ -** | | Total |
| --- | --- | --- | --- |
| **+** | TP | FP | (TP+FP) |
| **-** | FN | TN | (FN+TN) |

Sensitivity= TP/(TP+FN).

Specificity= TN/(TN+FP).

Accuracy= (TP+TN)/(TP+TN+FP+FN).

PPV= TP/(TP+FP).

NPV= TN/(TN+FN).

For the colorimetric RT-LAMP assay:

Sensitivity= [51/ (51+6)] *100 = 89.5%

Specificity= [105/ (105+3)] *100 = 97.2%

Accuracy= [(51+105)/ (51+105+3+6)] *100 = 94.5%

PPV= [51/ (51+3)] *100 = 94.4%

NPV= [105/ (105+6)] *100 = 94.6%

For the fluorometric RT-LAMP assay:

Sensitivity= [47/ (47+4)] *100 = 92.2%

Specificity= [98/ (98+1)] *100 = 99%

Accuracy= [(47+98)/ (47+98+4+1)] *100 = 96.7%

PPV= [47/ (47+1)] *100 = 98%

NPV= [98/ (98+4)] *100 = 96.1%
